# Supplementary material for: MetaQTL: a package of new computational methods for the meta-analysis of QTL mapping experiments
Source: BMC Bioinformatics. 2007 Feb 8;8:49. doi: 10.1186/1471-2105-8-49 (PMC1808479; doi:10.1186/1471-2105-8-49)
Supplement: Additional File 7 — MetaQTL Package : jar file and tutorial. This Zip archive contains both the MetaQTL JAR file and the files of the tutorial. [file 1471-2105-8-49-S7.zip › org.inra.metaqtl/doc/org/metaqtl/bio/class-use/IBioAdaptable.html]

Uses of Interface org.metaqtl.bio.IBioAdaptable


|  |  |  |
| --- | --- | --- |
| **Overview** \| **Package** \| **Class** \| **Use** \| **Tree** \| **Deprecated** \| **Index** \| **Help** | |  |
| PREV   NEXT | **FRAMES**    **NO FRAMES**     **All Classes** |  |


---


## **Uses of Interface org.metaqtl.bio.IBioAdaptable**

| Packages that use IBioAdaptable | |
| --- | --- |
| **org.metaqtl.bio** |  |
| **org.metaqtl.bio.entity** |  |

Uses of IBioAdaptable in org.metaqtl.bio

| Subinterfaces of IBioAdaptable in org.metaqtl.bio | |
| --- | --- |
| `interface` | `IBioAllele`             Class Description Here |
| `interface` | `IBioCross`             Class Description Here |
| `interface` | `IBioEntity`             Class Description Here |
| `interface` | `IBioGenome` |
| `interface` | `IBioIndividual`             Class Description Here |
| `interface` | `IBioLGroup` |
| `interface` | `IBioLocus` |
| `interface` | `IBioOntology`             This interface defines some rules to handle a ontology. |
| `interface` | `IBioOntologyTerm`             This class defines a term of an ontology. |
| `interface` | `IBioPopulation`             Class Description Here |

Uses of IBioAdaptable in org.metaqtl.bio.entity

| Classes in org.metaqtl.bio.entity that implement IBioAdaptable | |
| --- | --- |
| `class` | `AlleleContainer`             Class Description Here |
| `class` | `BioEntity`             Class Description Here |
| `class` | `BioEntityCollection`             Class Description Here |
| `class` | `BioEntityContainer`             Class Description Here |
| `class` | `CrossPopulation`             Class Description Here |
| `class` | `GeneticMap`             Class Description Here |
| `class` | `GroupContainer`             Class Description Here |
| `class` | `Individual`             Class Description Here |
| `class` | `IndividualContainer`             Class Description Here |
| `class` | `LGroup`             Class Description Here |
| `class` | `Locus` |
| `class` | `LocusContainer`             Class Description Here |
| `class` | `Marker`             Class Description Here |
| `class` | `Ontology` |
| `class` | `OntologyTerm` |
| `class` | `Population`             Class Description Here |
| `class` | `QTL`             Class Description Here |

---


|  |  |  |
| --- | --- | --- |
| **Overview** \| **Package** \| **Class** \| **Use** \| **Tree** \| **Deprecated** \| **Index** \| **Help** | |  |
| PREV   NEXT | **FRAMES**    **NO FRAMES**     **All Classes** |  |


---
